# Coacervate-mediated novel pancreatic cancer drug *Aleuria Aurantia* lectin delivery for augmented anticancer therapy

**DOI:** 10.1186/s40824-022-00282-6

**Published:** 2022-07-22

**Authors:** Sungjun Kim, Yunyoung Choi, Kyobum Kim

**Affiliations:** Department of Chemical & Biochemical Engineering, Dongguk University, Seoul, 04620 Republic of Korea

**Keywords:** Pancreatic cancer, Anticancer therapy, *Aleuria Aurantia* lectin, Coacervate, Drug delivery system

## Abstract

**Background:**

Pancreatic cancer, one of the cancers with the highest mortality rate, has very limited clinical treatment. Cancer cells express abnormal glycans on the surface, and some lectins with a high affinity for the glycans induce apoptosis in cancer. In this study, the efficacy of *Aleuria Aurantia* lectin (AAL) for the treatment of pancreatic cancer was evaluated and the efficacy improvement through AAL delivery with mPEGylated coacervate (mPEG-Coa) was investigated.

**Methods:**

AAL was treated with pancreatic cancer cells, PANC-1, and the expression level of caspase-3 and subsequent apoptosis was analyzed. In particular, the anticancer efficacy of AAL was compared with that of concanavalin A, one of the representative anticancer lectins. Then, methoxypolyethylene glycol-poly(ethylene arginylaspartate diglyceride), a polycation, was synthesized, and an mPEG-Coa complex was prepared with polyanion heparin. The AAL was incorporated into the mPEG-Coa and the release kinetics of the AAL from the mPEG-Coa and the cargo protection capacity of the mPEG-Coa were evaluated. Finally, improved anticancer ability through Coa-mediated AAL delivery was assessed.

**Results:**

These results indicated that AAL is a potential effective pancreatic cancer treatment. Moreover, mPEG-Coa rapidly released AAL at pH 6.5, an acidic condition in the cancer microenvironment. The initial rapid release of AAL effectively suppressed pancreatic cancer cells, and the continuous supply of AAL through the Coa transporter effectively inhibited proliferation recurrence of cancer cells.

**Conclusion:**

AAL is a potential novel drug for the treatment of pancreatic cancer therapeutic agent. In addition, a continuous supply of drugs above the therapeutic threshold using mPEG-Coa could improve therapeutic efficacy.

**Supplementary Information:**

The online version contains supplementary material available at 10.1186/s40824-022-00282-6.

## Background

Pancreatic cancer has a high mortality rate and the lowest overall survival of all the cancers [1]. Despite vigorous efforts in the advancement of diagnostic and therapeutic modalities in the recent decades, the overall 5-year survival rate for pancreatic cancer is less than 10% [2]. Since an efficient surgical treatment is not possible for most pancreatic ductal adenocarcinoma (PDAC) patients, single and combination chemotherapies using gemcitabine, Abraxane®, or FOLFIRINOX have been the only treatment options [3]. However, intravenous infusion of gemcitabine, which is the most common treatment option, could cause serious side effects including myelosuppression thrombocytopenia, anaemia, granulocytopenia, and neutropenia [4]. Therefore, there is an increasing need for new effective, and safe anticancer bio-therapeutics specific to pancreatic cancer.

Found ubiquitously in animals, plants, and fungi, lectins are carbohydrate-binding proteins that perform a variety of biological roles [5]. In particular, glycans on the surfaces of tumor cells influence tumor cell division, migration (metastasis), and recognition by the immune system [6]. Cancer cells exhibit a variety of altered glycosylation patterns in surface biomolecules such as glycolipids and glycoproteins, and the recognition of these aberrant changes may provide a basis for the discovery of biomarkers and therapeutic agents [7, 8]. Because of their ability to discriminate these variances in carbohydrate structures, plant lectins are potential diagnostic reagents and promising candidates for medicinal and clinical applications in many research fields [9]. Moreover, it is known that lectin-induced cancer cell surface glycan binding induces caspase-mediated apoptosis in cancer cells [10]. Consequently, several lectins with anticancer therapeutic properties have recently been spotlighted as natural anticancer agents. For instance, concanavalin A (Con A), a well-known anticancer lectin that induces caspase-mediated apoptosis in cancer cells, is effective against a wide range of cancers, including melanoma and colon cancer [11]. In addition, Schwarz et al. showed the inhibitory effect of Con A on the proliferation of pancreatic cancer. In our previous study, *Aleuria Aurantia* lectin (AAL), a fucose-targeting lectin, was shown to have a high surface binding ability to pancreatic cancer cells compared to plant lectins Con A and CA19–9 antibody [12]. Hence, in this study, AAL was selected as a promising drug candidate for pancreatic cancer treatment and its therapeutic efficacy was analyzed.

**Fig. 1:**
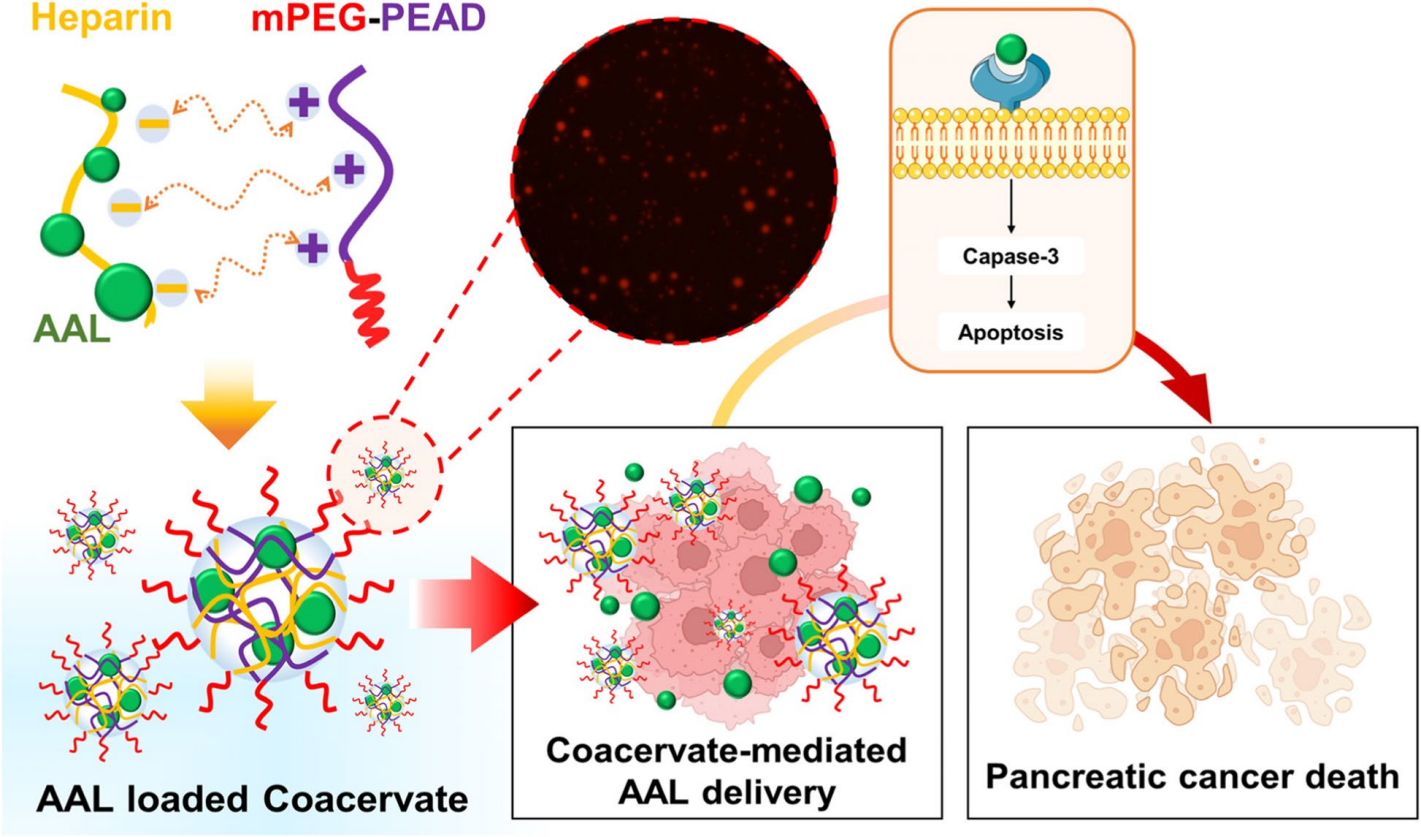
Illustration of Coa-mediated AAL delivery and subsequent augmented anticancer therapy against pancreatic cancer

Although biopharmaceuticals have exhibited excellent anticancer efficacy, a cargo drug directly administrated into a body could lose expected therapeutic functions due to short half-life, instability in blood circulation, enzymatic degradation, and immunogenicity [13]. Therefore, the development of a sustained-release exogenous delivery system that protects a therapeutic agent for a long period is necessary to increase therapeutic efficacy and efficiency. Polycation-based coacervates (Coa) can be excellent protein delivery carriers. A polycation methoxypolyethylene glycol-poly(ethylene arginylaspartate diglyceride) (mPEG-PEAD) forms a spherical droplet structure of mPEG-Coa in aqueous conditions through self-assembly with its polyanionic heparin counterpart via electrostatic interaction and following liquid-liquid phase separation [14, 15]. Coa can protect the incorporated cargo protein, preserve its bioactivity, and induce a sustained release of cargo molecules. In our previous studies, the therapeutic efficacy of Coa-mediated exogenous protein delivery was demonstrated in various applications including bone regeneration [16, 17], cartilage repair [18], angiogenesis [15], and skin wound healing [19, 20]. Moreover, a simple chemical modification (i.e., mPEGylation) of the cation backbone effectively improved the colloidal stability of Coa structure [14, 15]. Therefore, exogenous delivery of AAL using Coa could protect encapsulated cargo AAL from the harsh external environment while continuously releasing it, thereby improving their therapeutic efficacy (Fig. 1).

In the present study, we hypothesized that AAL lectin, which has a high affinity for pancreatic cancer surface glycans, is a superior treatment for pancreatic cancer, especially with the aid of mPEG-Coa for sustained AAL delivery and cargo protection. Consequently, a continuous supply of AAL with preserved bioactivity could achieve excellent anticancer effects. The current study investigated (1) anticancer efficacy of AAL, (2) Coa-mediated protective ability and sustained release kinetics of cargo AAL, and (3) enhanced anti-pancreatic cancer agents’ efficacy through exogenous AAL delivery.

## Methods

### Materials

AAL, biotinylated AAL (biotin-AAL), and Con A were purchased from Vector Laboratories. Dulbecco’s Modified Eagle Medium (DMEM), penicillin–streptomycin, fetal bovine serum (FBS), trypsin, and phosphate-buffered saline (PBS) were obtained from Corning. Keratinocyte-SFM and CellTracker™ Red CMTPX Dye were received from Invtrogen. Caspase-3 assay kit, Cell Meter™ Live Cell TUNEL Apoptosis assay Kit, EZ-Cytox were purchased from BioVision, AAT Bioquest, and DoGenBio, respectively. Fmoc-Asp and Fmoc-Arg were obtained from BOC Science. Monomethoxy polyethylene glycol 350, 1-ethyl-3-(3-dimethylaminopropyl) carbodiimide (EDC), N-hydroxy succinimide (NHS), tetrabutylammonium bromide (TBAB), 4-dimethyl amino pyridine (DMAP), dimethyl formamide (DMF) (anhydrous), 1,4-dioxane, n-Hexane, and piperidine were purchased from Sigma-Aldrich. Ethylene glycol diglycidyl ether (EGDE), heparin (molecular weight: approximately 6000 Da), protease inhibitor, and Bacteroides Heparinase II were obtained from TCI, Selleckchem, Thermo Fisher Scientific, and New England Biolabs, respectively.

### Comparison of anticancer efficacy between AAL and Con A

Pancreatic cancer cell line PANC-1 was cultured in a basal medium consisting of DMEM (89% (v/v)), penicillin–streptomycin solution (1% (v/v)), and FBS (10% (v/v)). When the confluency reached approximately 80%, PANC-1 cells were trypsinized, seeded at 100,000 cells/well on 24-well cell culture plates, and incubated at 37 °C, 5% CO_2_, and 95% humidity for 24 h. After 24 h, the old media was discarded and changed to fresh media containing 100 μg/mL of Con A or 100 μg/mL of AAL and incubated for 12 h. To compare the caspase-3 inducing ability between AAL and Con A, we performed a Caspase-3 assay according to the manufacturer’s suggestion. In addition, to analyze the level of caspase-mediated apoptosis, a TUNEL assay was carried out according to the manufacturer’s protocol.

### Pancreatic cancer-specific anticancer effects of AAL

PANC-1 cells were cultured as mentioned above and normal human pancreatic ductal epithelial cells H6C7 (Korean Cell Line Bank, Seoul, Republic of Korea, passage number 7) were cultured in keratinocyte-SFM (serum-free medium) with supplements. Each cell line was seeded at 10,000 cells/well on 96-well cell culture plates, and incubated at 37 °C, 5% CO_2_, and 95% humidity for 24 h. After 24 h, to analyze the pancreatic cancer-specific anticancer efficacy by the concentration of AAL, the old media was replaced with a medium containing 0–100 μg/mL of AAL and incubated for 24 h. After 24 h of incubation, cell viability was determined by WST-1 assay. WST-1 reagent was prepared by mixing EZ-Cytox with basal media at a 1:10 (v/v) ratio, treated to each well, and incubated for 3 h. Subsequently, optical density was measured at 440 nm by a microplate spectrophotometry.

### Synthesis of mPEG-PEAD

First, 5 mmol of Fmoc-Asp, 5 mmol of EGDE, and 5 mg of TBAB were dissolved in 2.5 mL of 1,4-dioxane, and polymerization was done at 100 °C for 48 h. Obtained intermediate polymer, poly(ethylene Fmoc-aspartate diglyceride) (FPED), was washed using diethyl ether and dried under vacuum conditions. After that, the FPED was perfectly dissolved in 5 mL of DMF. Subsequently, 1 mmol of EDC, 1 mmol of NHS, and 5 mg of DMAP were mixed in FPED solution for 1 h. The number average molecular weight (Mn) and polydispersity index (PDI) of FPED were measured by Agilent gel permeation chromatography (GPC) system with Styragel GPC column (Waters) and 0.1% LiBr in DMF as a mobile phase at a flow rate of 1 mL/min. Then, 1 mmol of mPEG (MW 350) dissolved in 2 mL of DMF was applied to the mixture, and the reaction was continued for 48 h. After 48 h, 4 mL of piperidine was added and reacted for removing Fmoc moiety. Deprotected mPEG-PED was precipitated by the addition of diethyl ether, washed by diethyl ether three times, and dried under vacuum conditions. Next, 5 mmol of Fmoc-Arg was dissolved in 10 mL of DMF with 5 mmol of EDC, 5 mmol of NHS, and 5 mg of DMAP and stirred for 30 min. Additionally, mPEG-PED was dissolved in 5 mL of DMF, and a prepared Fmoc-Arg mixture was added to mPEG-PED solution. The reaction was accomplished for 48 h. Then, 4 mL of piperidine was mixed and reacted for 30 min. mPEG-PEAD was precipitated by adding diethyl ether, washed 3 times with diethyl ether, 1 time with hexane, 1 time with diethyl ether, and dried under vacuum. Finally, mPEG-PEAD was dissolved in 50 mL of 0.1 M HCl solution, dialyzed using a dialysis tube (MWCO = 2000) against DW for 24 h, and lyophilized. Polymer structures were characterized via ^1^H-NMR instrument.

### Zeta potential measurement

The mPEG-PEAD and heparin were dissolved in DW at a concentration of 10 mg/mL, respectively. Prepared mPEG-PEAD was mixed with 5 μL of fixed heparin solution at different weight ratios: 2:1, 5:1, 7:1, 7.5:1, 8:1, 10:1, 15:1, 20:1, and 30:1. Additionally, DW was added to the mPEG-PEAD/heparin mixture to achieve a total volume of 700 μL. Zeta potential was measured using a Malvern Zetasizer. The isoelectric point (8:1 of mPEG-PEAD:heparin mass ratio) was used to prepare Coa in subsequent studies.

### Fluorescence imaging of coacervates

For fluorescence imaging of Coa, a fluorescent dye, CellTracker Red, was incorporated into Coa. First, 4 μL of CellTracker Red was mixed with 50 μL of heparin solution (10 mg/mL), and then 400 μL of mPEG-PEAD was added. Supernatant was removed after centrifuging at 15,000 rpm for 15 min and resuspended with 200 μL of pH 7.4 PBS or pH 6.5 PBS. Coa complexes were observed using a fluorescence microscope (Nikon Ti-E) at different time intervals: 5-, 30-, and 60-min. Circularity (i.e., Colloidal stability) of Coa was estimated using randomly selected 30 particles by ImageJ software (http://rsbweb.nih.gov/ij/, National Institutes of Health, Bethesda, MD, USA).

### Preparation of AAL loaded coacervates

10 mg/mL of mPEG-PEAD and heparin were separately prepared in PBS and sterilized using a 0.22 µm filter membrane. Then, 2 μL of AAL (0.5 mg/mL) was first mixed with 10 μL of heparin solution. Thereafter, 80 μL of mPEG-PEAD solution was added to the heparin:AAL mixture. The final mass ratio was 1:100:800 of AAL:heparin:mPEG-PEAD. An increase in the solution turbidity was immediately observed due to the instantaneous formation of Coa. Particle size of fabricated AAL loaded mPEG-Coa or empty mPEG-Coa (i.e., without AAL loading) was measured by Malvern Zetasizer.

### In vitro AAL release kinetics

Herein, biotin-AAL was used to quantify unloaded and released AAL by reaction with streptavidin-HRP and substrate TMB. AAL loaded Coa was prepared as previously described. Each group containing 1 μg of biotin-AAL was placed in pH 7.4 PBS or pH 6.5 PBS and incubated at 37 °C for 21 days. After centrifuging at 15,000 xg for 15 min on days 0, 1, 2, 3, 5, 7, and 14, the supernatant was collected and replaced with PBS of the same pH. Day 0 sample was used to calculate AAL loading efficiency. Unloaded and released AAL in collected supernatants was measured using a custom ELISA-like assay. One hundred microliters of collected samples were transferred to a 96-well ELISA plate and incubated for 24 h. After adsorption of the lectins, the coated plates were blocked with 1% BSA, after washing with PBST. Then, 100 μL of avidin-HRP (25 ng/mL) was applied and incubated for 30 min. After incubation and washing, 100 μL of ABTS substrate solution was added to each well, incubated for 30 min, and the optical density was measured by a microplate spectrophotometry at 405 nm with wavelength correction set at 650 nm. AAL amount was calculated using a biotin-AAL standard curve.

### Cargo AAL protection ability of coacervates in trypsin

Considering the loading efficiency, Coa containing 1 μg of AAL was prepared. AAL loaded Coa or bolus AAL was incubated in 100 μL of 500 ng/mL trypsin for 0, 12, or 24 h. At each predetermined time point, 100 μL of 1× protease inhibitor solution was added and incubated for 5 min to exclude further digestion by trypsin. Then, 50 μL of heparinase cocktail solution (1 μL of 4000 units/mL) was applied and incubated at 37 °C for 2 h for complete disruption of Coa complex. The remaining AAL was directly determined using ELISA-like lectin quantification method described above in the section ‘In vitro AAL release kinetics’.

### Enhanced anticancer efficacy by coacervate-mediated AAL delivery

PANC-1 cells were seeded at 10,000 cells/well on 96-well cell culture plates, and incubated at 37 °C, 5% CO_2_, and 95% humidity for 24 h. After 24 h, the old media was removed and 100 μL of fresh media containing following components was added; empty mPEG-Coa (i.e., 100 μg of heparin and 800 μg of mPEG-PEAD complex), bolus AAL (1 μg), or AAL (1 μg)-loaded mPEG-Coa. After 1, 3, and 5 d, the media was removed from each well and WST-1 assay was performed.

### Statistical analysis

All quantitative data were expressed as mean standard deviation and analyzed by one-way analysis of variance (ANOVA) with Tukey’s *post-hoc* method, using Graphpad Prism V 7.0 (Graphpad Software Inc., San Diego, CA, USA). Differences were considered statistically significant when the *p*-value < 0.05.

## Results

### Anticancer efficacy of AAL

**Fig. 2:**
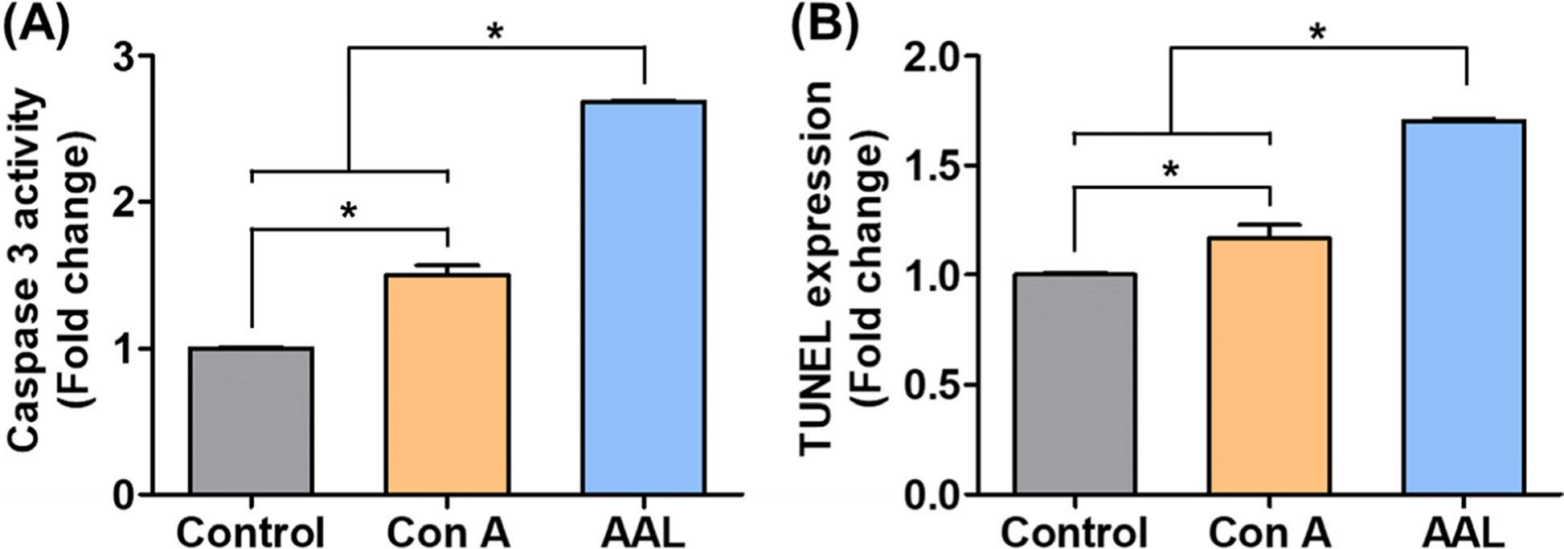
Anticancer mechanism of AAL against pancreatic cancer cell PANC-1. **A** Caspase-3 activity and **B** TUNEL expression (i.e., apoptosis) treated with AAL or Con A after 12 h

Caspase-3 induction by AAL treatment and corresponding apoptosis of pancreatic cancer cells were analyzed and compared to that of Con A (Fig. 2). When AAL was treated with pancreatic cancer cells PANC-1, the expression of caspase-3 was increased in the pancreatic cancer cells (Fig. 2A). In this regard, it could be carefully inferred that AAL, like other therapeutic lectins, triggered the downstream signaling pathways of caspase-3-mediated apoptosis including cytochrome C, caspase-9, and pro-caspase-3 [10, 21]. Consequently, the expression of TUNEL, an indicator of apoptosis increased (Fig. 2B). In addition, AAL effectively induced caspase-mediated pancreatic cancer apoptosis when compared with Con A.

**Fig. 3:**
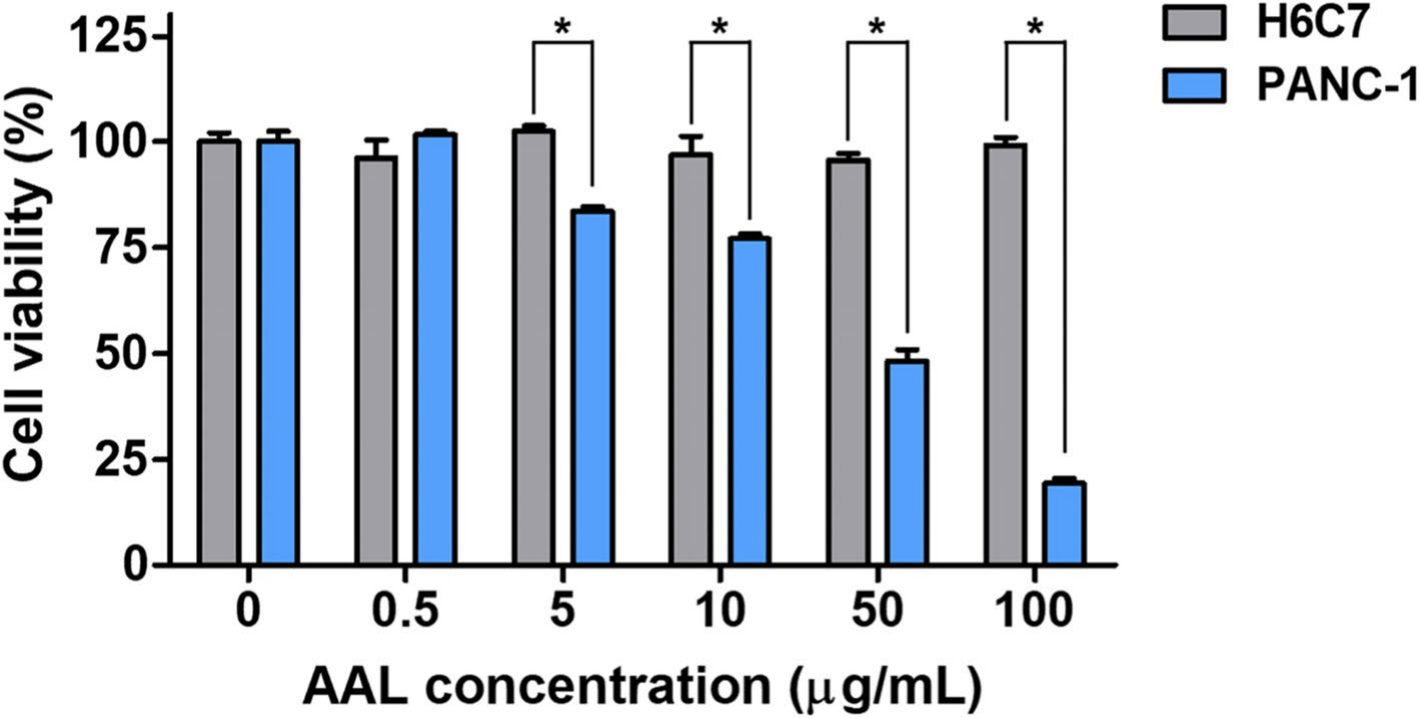
Biocompatibility of AAL to normal pancreas cell H6C7 and AAL concentration-dependent anticancer efficacy

The biocompatibility of AAL was also evaluated by investigating it’s in vitro cytotoxicity in normal pancreas cells H6C7 (Fig. 3). Up to 100 μg/mL of AAL exhibited excellent biocompatibility toward H6C7 cells. On the other hand, concentration-dependent cytotoxicity was observed in PANC-1. When 100 μg/mL of AAL was applied to the cells for 24 h, 99.2 ± 3.2% cell viability was observed in H6C7 cells, whereas only 19.3 ± 1.9% cell viability was detected in PANC-1.

### Characterization of mPEG-PEAD

**Fig. 4:**
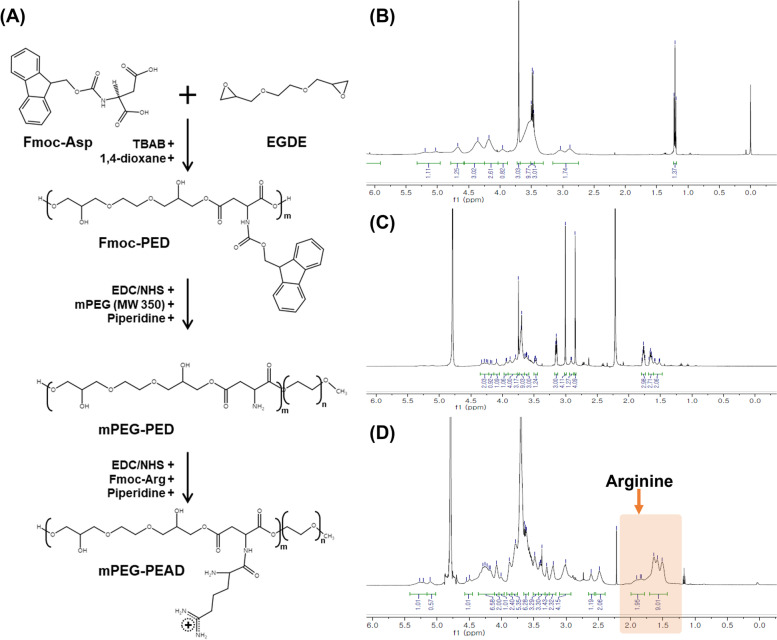
Synthesis and characterization of mPEG-PEAD. **A** General synthesis step of mPEG-PEAD and **B**-**D** structural characterization via ^1^H-NMR. **B** Fmoc-PED was prepared in CDCl_3_, and **C** mPEG-PED and **D** mPEG-PEAD were prepared in D_2_O

mPEG-PEAD polycation was synthesized according to the procedure described in Fig. 4A. Additionally, sequential chemical modification of the synthesized intermediates was analyzed using ^1^H-NMR spectral techniques (Fig. 4B-D). ^1^H-NMR spectrum of Fmoc-PED exhibited a signal at 5.0–5.4 (−CH proton of aspartate) and 3.2–4.6 ppm (methylene protons of glyceride moiety) (Fig. 4B). ^1^H-NMR spectrum of mPEG-PED showed a signal at 3.2 ppm (−CH_3_ group of mPEG) with 1.4–1.6 ppm (−NH_2_ of in the PED polymer side chain) (Fig. 4C). In addition, ^1^H-NMR spectrum of the final mPEG-PEAD polycation indicated signals at 1.3–1.8 ppm (conjugated arginine moiety) (Fig. 4D). Overall NMR spectral results revealed that the stepwise synthesis of mPEG-PEAD was successfully preceded. In addition, the number average molecular weight of FPED was 4559.3 g/mol with 1.5 PDI. Based on this results, degree of polymerization was calculated to approximately 8.6.

**Fig. 5:**
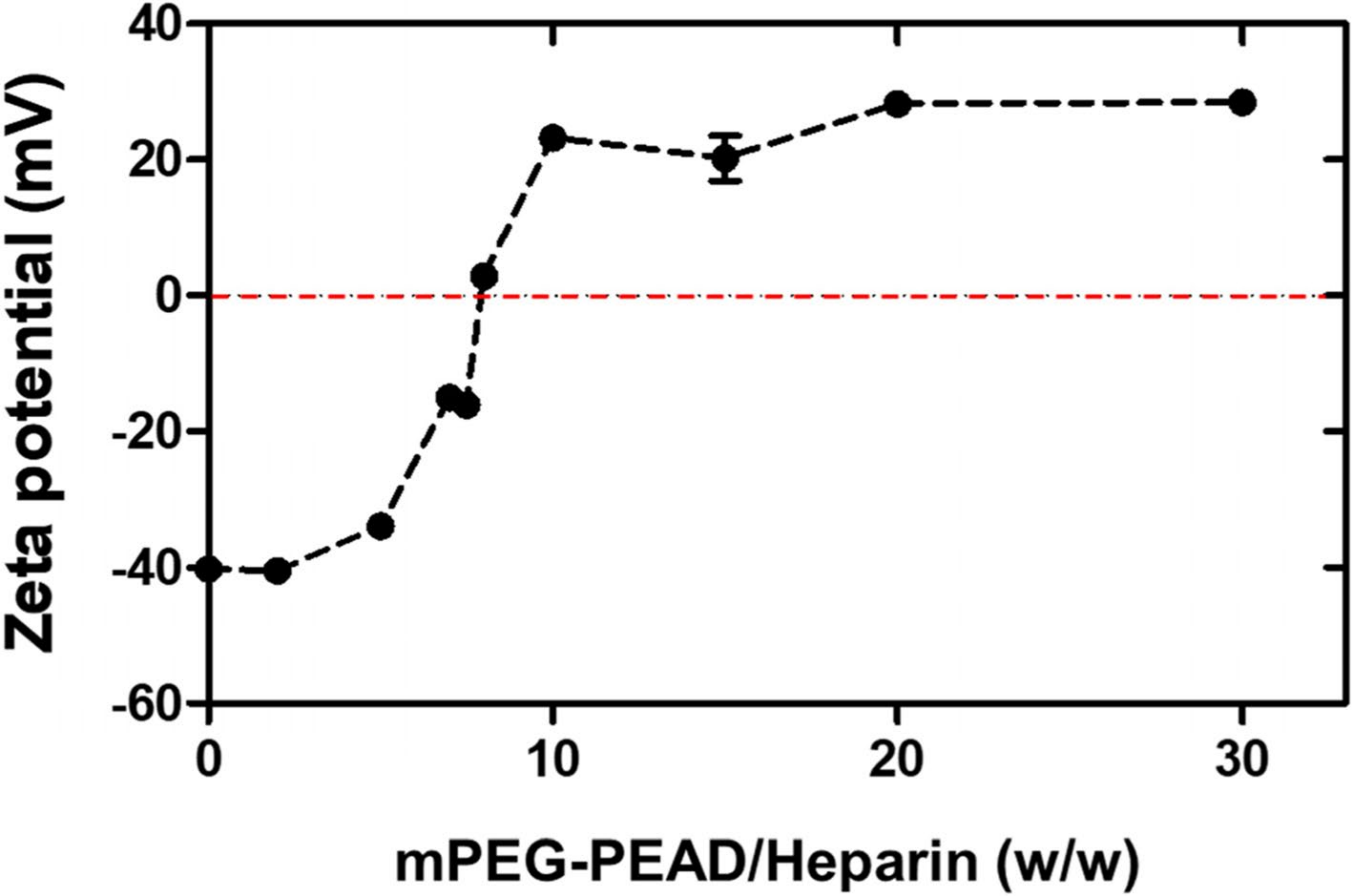
Zeta potential of mPEG-PEAD/heparin at different weight ratios

The synthesized mPEG-PEAD polycation had a zeta potential of approximately 26.7 mV. In order to determine the neutral condition of Coa complex during electrostatic interaction and subsequent self-assembly, zeta potentials at varying weight ratios of mPEG-PEAD/heparin were measured (Fig. 5). 8:1 of mass ratio indicated a neutral charge property of fabricated mPEG-Coa. Moreover, the particle size of mPEG-Coa slightly increased from 304.3 nm in empty mPEG-Coa (i.e., without AAL loading) to 491.0 nm after AAL loading into the mPEG-Coa (Fig. S1).

### Sustained release and cargo protection abilities of coacervate

**Fig. 6:**
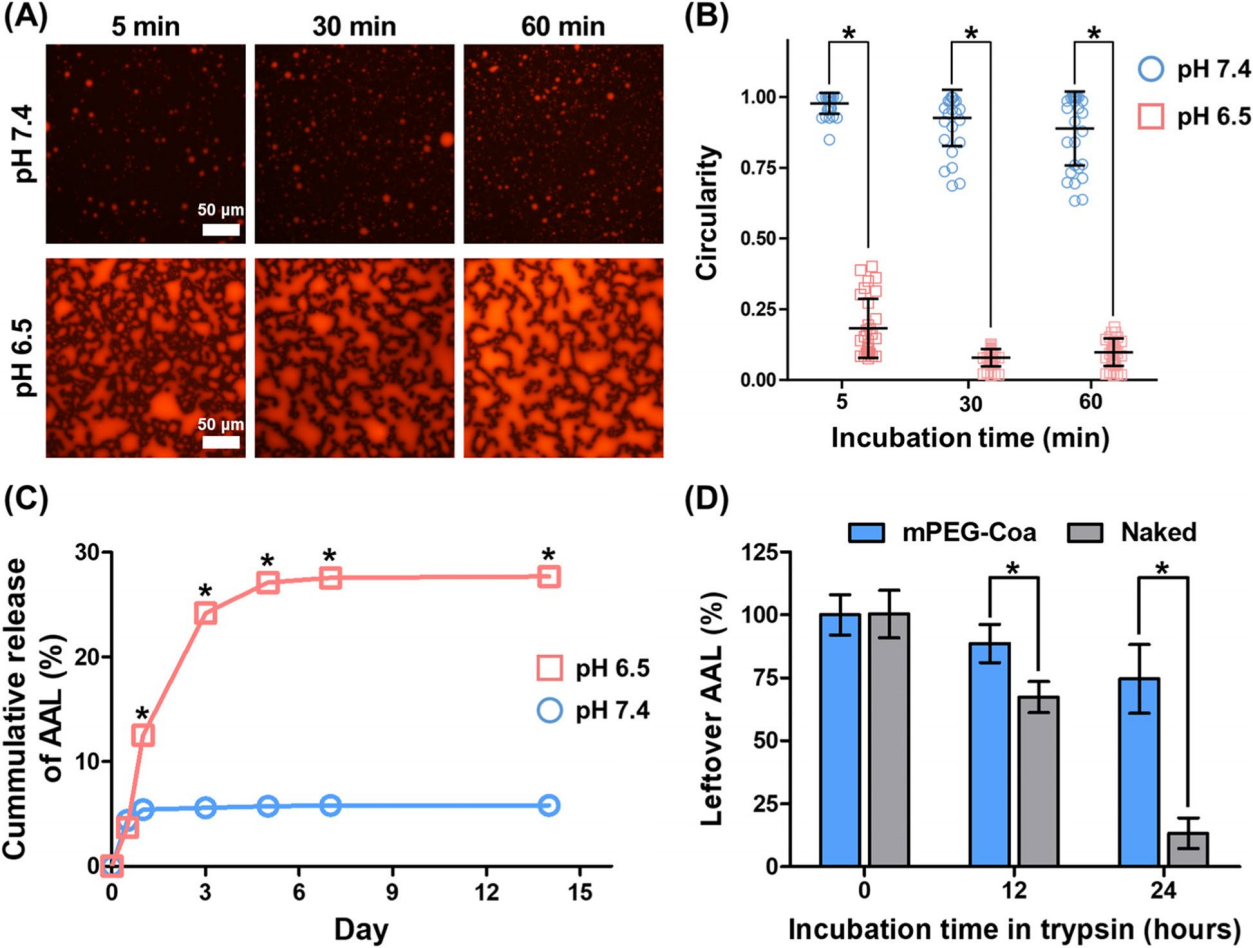
pH responsive **A** structural deformation of Coa and **B** circularity of Coa particles. **C** % cumulative cargo AAL release profile at different pH environment and **D** cargo AAL protection ability of Coa against protease

Colloidal stability and pH-responsive structural deformation of mPEG-Coa were evaluated by morphological transformation. Fluorescence images revealed that the formed mPEG-Coa maintains a stable spherical structure at pH 7.4 (Fig. 6A and B). On the other hand, an immediate deformation and random aggregation of mPEG-Coa structure were observed in an acidic environment (pH 6.4) (Fig. 6A and B).

Loading efficiency and release kinetics of cargo AAL in mPEG-Coa were evaluated by a custom ELISA-like lectin quantification assay. The initial loading efficiency of AAL into mPEG-Coa was over 99%. Although similar cargo AAL release rates were observed in both pH conditions on the first day, a burst release profile was observed in pH 6.5 thereafter (Fig. 6C). The mPEG-Coa in pH 7.4 exhibited a cumulative AAL release of 5.82 ± 0.15% for 14 days, whereas 27.68 ± 1.04% of release was detected in pH 6.5.

In addition to sustained delivery of cargo protein, mPEG-Coa mediated exogenous delivery offers effective cargo protection. To evaluate the cargo protection efficacy, AAL-loaded mPEG-Coa and naked bolus AAL were incubated in trypsin, a global protease. Significantly improved protection against a protein degradation enzyme in the surrounding environment was observed in encapsulated AAL in mPEG-Coa after 12 and 24 h at 37 °C (Fig. 6D). In contrast, in the naked AAL group, 67.33 ± 9.45% and 13.33 ± 6.11% of the initial AAL remained after 12 and 24 h of incubation, respectively.

### Enhanced anticancer efficacy via coacervate mediated AAL delivery

**Fig. 7:**
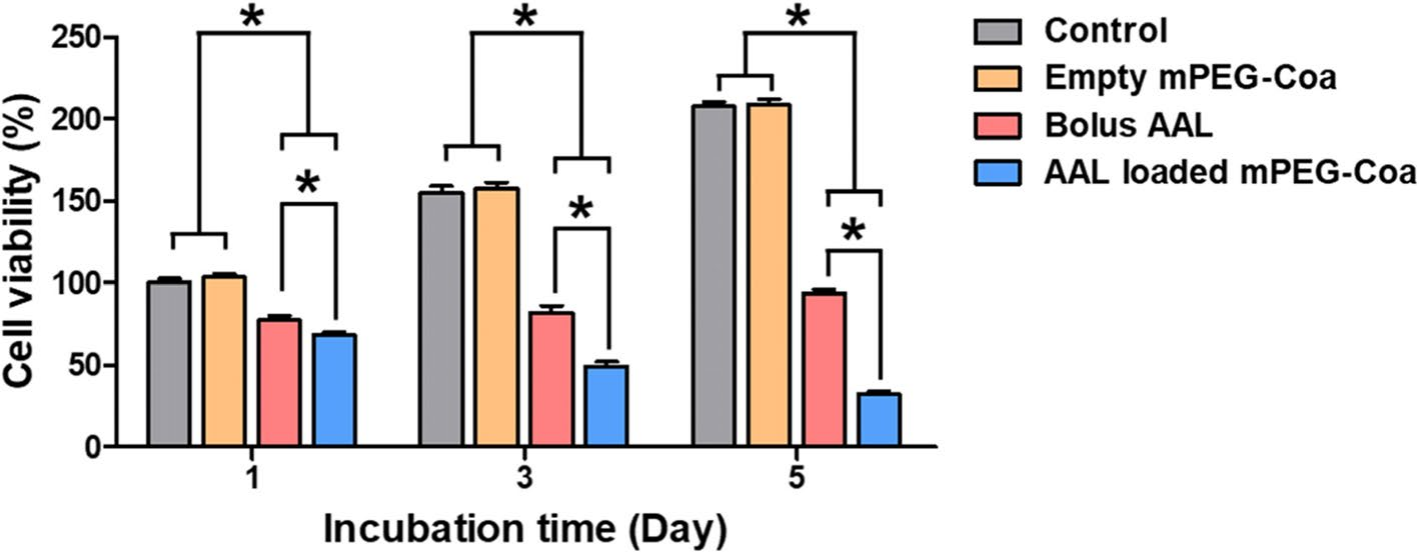
In vitro anticancer efficacy of AAL loaded coacervate on pancreatic cancer PANC-1 cells

Herein, PANC-1 cells were incubated with empty mPEG-Coa, bolus AAL, or AAL loaded mPEG-Coa for 5 days (Fig. 7). Treatment with empty mPEG-Coa did not induce any toxicity to PANC-1 for 5 days, indicating that the electrolyte components in mPEG-Coa had good biocompatibility. In addition, 77.2% of cell viability was observed on day 1 when PANC-1 was directly treated with bolus AAL. Although there was a higher cancer cell death with the treatment of bolus AAL than the control in all-time points, cancer cells slightly regrowth from day 1. Specifically, the viability of PANC-1 cells increased to 82.1 and 93.6% on days 3 and 5, respectively. On the other hand, in the AAL-loaded mPEG-Coa group, the viability of PANC-1 was significantly downregulated when compared with all the other groups and the survival rate of cancer cells continued to decrease until day 5. Specifically, 68.1, 49.3, and 32.0% of cell viabilities were observed in AAL loaded mPEG-Coa treated PANC-1 cells on days 1, 3, and 5, respectively.

## Discussion

Several plant lectins induce caspase-mediated apoptosis (i.e., programmed cell death) of cancer cells through interaction with sugar-binding receptors on the plasma membrane [22]. For instance, the following plant lectins have been used as caspase-mediated apoptosis for anticancer therapies: mistletoe lectin I for leukemia [23], Korean mistletoe for liver cancer [24] and squamous cell carcinoma [25], Dioclea violacea for glioma [26], and Polygonatum odoratum lectin for melanoma [27]. Con A is one of the representative anticancer lectins [28], which has been used for various anticancer treatments including breast cancer [29], melanoma [30], and hepatoma [28]. Activated caspase (a cysteine protease) could catalyze the cleavage of essential cellular proteins and trigger apoptosis [31]. In particular, since caspase-3 executed this cell death modality, we preferentially selected and tested caspase-3 as the main signaling molecule [32]. Accordingly, caspase-3 activity and subsequent apoptosis level by direct AAL treatment were determined in PANC-1 cells (Fig. 2). On the other hand, AAL was no toxic to normal pancreas cell H6C7 (Fig. 3). These results are closely related to the attraction of lectins to glycans on the cancer cell membranes. In our previous study, we demonstrated lectin-type dependent cellular surface affinities; (1) AAL showed a higher interaction with PANC-1 than Con A lectin, and (2) the affinity level of AAL to PANC-1 was also higher than in the other cell lines including H6C7, MDA-MB-231, HeLa, and human dermal fibroblasts [12]. In this regard, AAL is a potential candidate for a pancreatic cancer-specific therapeutic lectin with suitable safety for normal pancreas cells. To the best of our knowledge, this study was the first to investigate the anticancer properties of AAL lectin. Thus, sufficient follow-up studies on the in vivo safety and anticancer mechanism of AAL should be conducted to further confirm AAL as a successful anticancer agent and its mechanism of action.

Although direct treatment of AAL induced apoptosis in pancreatic cancer cells, administration of the protein without a delivery carrier could not achieve the expected therapeutic effect due to their short half-life [13]. Therefore, we synthesized mPEG-PEAD (Fig. 4) and fabricated mPEG-Coa as a AAL delivery carrier using polycation mPEG-PEAD and polyanion heparin. Here, PEGylation to polycations provides steric stability to the coacervate complex in a harsh environment [33] and their colloidal stability in aqueous conditions could be improved [14, 15]. The enhanced stability of mPEG-Coa by PEGylation and its usefulness as an excellent growth factor carrier were demonstrated in our previous study, which suggested that the optimal range of PEG chain length for cation modification (MW 350) should be considered in ionic environments. In addition, our mPEG-Coa showed excellent AAL loading efficiency of more than 99%. Similar high cargo loading efficiencies of over 96% were observed in our previous studies using mPEG-Coa for bone morphogenetic protein-2 [14] and vascular endothelial growth factor [15]. Thus, our mPEGylated PEAD-based Coa could be an effective AAL delivery platform with augmented colloidal stability. Moreover, the fabrication with 8:1 of mPEG-PEAD:heparin mass ratio (Fig. 5) was performed in subsequent studies, as the neutral surface charge of mPEG-Coa complex potentially prevents undesired non-specific adsorption of serum proteins, especially in a physiological environment, and further reduces the interference in diffusional release of cargo proteins in mPEG-Coa. The enhanced structural stability by mPEGylation was also demonstrated in our previous study. In this previous study, control Coa without PEG modification showed a randomly aggregated shape after 15 min of incubation in 0.3 M NaCl solution, whereas mPEG-Coa maintained its colloidal morphology for more than 30 min [14]. Similarly, our Coa also retained its spherical shape at neutral pH, whereas its structure immediately deformed and randomly aggregated in an acidic environment (Fig. 6A and B). During the coacervation driven by electrostatic interactions between two polyelectrolytes (i.e., mPEG-PEAD cation and heparin), the change in the ionization degree of the components strongly affected the mutual affinity of the components, resulting in phase separation or transformation [34]. Therefore, mPEG-PEAD and heparin exposed to an acidic environment are protonated and the dissociation of mPEG-Coa complex commences. A similar phenomenon of the pH-dependent structural disruption was also observed in our previous study [35]. Here, cationic polymer poly(3-arginylamino propylene succinate-b-polypropylene glycol)-mediated Coa maintained its stable spherical shape for 24 h in a neutral environment, while dissociation of polyelectrolytes and subsequent deformation of Coa was observed within 1 h in an acidic environment (pH 5.0). It has been reported that the release of cargo proteins from PEAD-based Coa dominantly depends on (1) the dissociation constant (Kd) between heparin and cargo protein molecule and (2) hydrolysis of PEAD cation backbone [36]. Therefore, in acidic pH conditions, the modulated surrounding ionic strength and further protonation of mPEG-PEAD and heparin results in sequential dissociation of electrolytes, deformation of mPEG-Coa structure (Fig. 6A and B), and accelerated release of cargo AAL (Fig. 6C). In particular, the pH-dependent cargo release profile indicated that our mPEG-Coa-mediated drug delivery system could burst and release the cargo AAL in the acidic tumor microenvironment (TME). In addition, the mPEG-Coa could not only regulate the sustained release profile of the cargo AAL, but also protect the cargo from the harsh external environment. After 24 h of trypsin exposure, 74.7% of the AAL remained when loaded on mPEG-Cao, whereas only 13.3% remained in the naked AAL (Fig. 6D). Cargo protection ability of Coa has also been demonstrated in previous studies [37, 38]. For instance, when fibroblast growth factor-2 (FGF2) was loaded into Coa and exposed to trypsin, no degradation of cargo FGF2 was observed for 2 h, whereas naked FGF2 was completely degraded [37]. Moreover, after 10 h exposure of naked interleukin-12 (IL-12) to trypsin, only 2.1% of IL-12 was remained, whereas 70.1% of cargo IL-12 remained when loaded in Coa [38]. Furthermore, it has been also demonstrated that Coa-based delivery platform could not only prevent the degradation of cargo proteins from foreign enzymes, but also maintain the structure/functionality of proteins [18, 38]. In this study, since splenocytes secrete IFN-ϒ in response to IL-12, released IL-12 from Coa was collected and treated to mouse splenocyte. As a result, there was no significant difference in IFN secretion between the released IL-12-treated splenocyte group and the fresh IL-12-treated group, indicating that there was no effect on the functionality of released IL-12. In addition, released insulin-like growth factor-1 (IGF-1) from Coa, which embedded in 3D hydrogel was collected and treated with MCF-7 cells [18]. As a result, the growth of MCF-7 was promoted at the similar level as the group treated with fresh IGF-1. On the other hand, when bolus IGF-1 was directly loaded into the hydrogel (without coacervate-mediated protection), the promoted growth rate of MCF-7 cells by released bolus IFG-1 was not observed, as compared to the fresh IGF-1 treated group. These results demonstrated that our mPEG-Coa-based AAL delivery system could (1) protect incorporated cargo AALs from enzymatic degradation and sufficiently preserve their therapeutic activity and (2) induce pH-dependent AAL release, especially in acidic conditions (i.e., pH 6.5–6.8) of TME [39].

Finally, augmented anticancer efficacy of mPEG-Coa-mediated long-term AAL delivery, against pancreatic cancer cells, was investigated (Fig. 7). Here, empty Coa had no effect on PANC-1 cells for 5 days. Although cationic polymer-based delivery systems have been actively developed in recent decades, their toxicity hampers the clinical translation for tissue regeneration and disease treatment [36, 40]. While the deleterious effects of polycations could damage cell membranes and induce apoptosis [41, 42], suitable in vitro and in vivo biocompatibility of PEAD-based Coa has been demonstrated through a series of studies [14, 15, 36]. Interestingly, the administration of bolus AAL provided a more abundant dose of available AAL in the surrounding environment during the initial interaction with cancer cells, but facilitated cancer cell death was detected in mPEG-Coa-mediated AAL treatment. Moreover, viability of bolus AAL treated PANC-1 cells was gradually increased from days 1 (77.2% of viability) to 5 (93.6% of viability), indicating a recurrence proliferation of PANC-1 cells. However, the treatment using released AAL from mPEG-Coa effectively suppressed the proliferation of PANC-1 cells up to 32% on day 5. When the cargo protein is released from Coa by hydrolysis of PEAD backbone, it is most likely in the form of a cargo:heparin complex, where heparin could stabilize and protect the cargo protein and induce the binding of the protein to its receptor [38, 43]. Therefore, the protein released from Coa could reveal better bioactivity than the freshly prepared bolus protein. For instance, interleukin-12 (IL-12) released from Coa exhibited improved bioactivity of up to 762.7% compared to the same amount of fresh IL-12 [38]. As compared to the inactivation of bolus AAL (i.e., without cargo protection) and subsequently reduced apoptosis-mediated PANC-1 cancer cells, released cargo AAL from mPEG-Coa (1) efficiently maintained its bioactivity via the protection efficacy of carriers and (2) effectively induced PANC-1 death, even with the less available therapeutic dose (i.e., 5.4, 5.6, and 5.7% cumulative release in pH 7.4 and 12.5, 24.2, and 27.1% in pH 6.5 on day 1, 3, and 5 in Fig. 6C) than directly applied bolus AALs. Therefore, rapid release of cargo AAL from mPEG-Coa by pH in TME could successfully induce early cancer cell inhibition. The late gradual release profile of AAL from mPEG-Coa also demonstrated the importance of a continuous supply of therapeutically available AAL doses above the threshold for anticancer treatment and prevention of proliferation recurrence.

## Conclusion

In this study, we evaluated the anticancer efficacy of AAL, a novel protein drug candidate for the treatment of pancreatic cancer. AAL induced caspase-3 mediated apoptosis of PANC-1 cells with an efficacy that was higher than one of the representative anticancer Con A. In addition, AAL induced pancreatic cancer cell death in a concentration-dependent manner without affecting normal pancreas cells. Moreover, in this study, the modulated coacervate complex by mPEGylation into a polycation component (i.e., mPEG-Coa) could protect the incorporated cargo AALs from the surrounding environment and exhibited pH-responsive early burst and late gradual release kinetics, especially in acidic pH in TME. Subsequently, continuously supplied AALs above the therapeutic threshold from mPEG-Coa effectively suppressed the proliferation of pancreatic cancer cells. However, since this present study was the first trial of AAL-based cancer treatment, a follow-up study on the anticancer mechanism and the clinical safety of cargo AAL should be further performed. Nevertheless, it could be reasonably concluded that (1) AAL is a potentially effective treatment for pancreatic cancer and (2) continuous supply of this lectin-based anticancer agent through mPEG-Coa-mediated exogenous delivery could significantly inhibit cancer progress and proliferation recurrence. Furthermore, our previous results in enhanced cellular activities by delivering a variety of proteins/cytokines demonstrated the mPEG-Coa as an effective carrier that can improve therapeutic functionalities of not only AAL but also various conventional anticancer and protein drugs.

## Supplementary Information

**Additional file 1: Figure S1.** Size distribution of empty mPEG-Coa and AAL loaded mPEG-Coa.

## Data Availability

For data requests, please contact the authors.

## Abbreviations

AAL: *Aleuria Aurantia* lectin
Biotin-AAL: Biotinylated AAL
Coa: Coacervate
Con A: Concanavalin A
DMAP: 4-dimethyl amino pyridine
DMEM: Dulbecco’s Modified Eagle Medium
DMF: Dimethyl formamide
EDC: 1-ethyl-3-(3-dimethylaminopropyl) carbodiimide
EGDE: Ethylene glycol diglycidyl ether
FBS: Fetal bovine serum
FPED: Poly(ethylene Fmoc-aspartate diglyceride)
mPEG-Coa: mPEGylated coacervate
mPEG-PEAD: Methoxypolyethylene glycol-poly(ethylene arginylaspartate diglyceride)
NHS: N-hydroxy succinimide
PBS: Phosphate-buffered saline
PDAC: Pancreatic ductal adenocarcinoma
TBAB: Tetrabutylammonium bromide
TME: Tumor microenvironment
