# Supplementary material for: Coacervate-mediated novel pancreatic cancer drug Aleuria Aurantia lectin delivery for augmented anticancer therapy
Source: Biomater Res. 2022 Jul 22;26:35. doi: 10.1186/s40824-022-00282-6 (PMC9308356; doi:10.1186/s40824-022-00282-6)
Supplement: Supplementary file 1 — Additional file 1: Figure S1. Size distribution of empty mPEG-Coa and AAL loaded mPEG-Coa. [file 40824_2022_282_MOESM1_ESM.docx]

Supporting information

**Coacervate-mediated novel pancreatic cancer drug *Aleuria Aurantia* Lectin delivery for augmented anticancer therapy**

Sungjun Kim^1^, Yunyoung Choi^1^, and Kyobum Kim^1,*^

^1^Department of Chemical & Biochemical Engineering, Dongguk University, Seoul, Republic of Korea

*Corresponding Author: Kyobum Kim, Ph.D.

Associate Professor

Department of Chemical & Biochemical Engineering

Dongguk University, Seoul 04620, Korea

Email: kyobum.kim@dongguk.edu

**
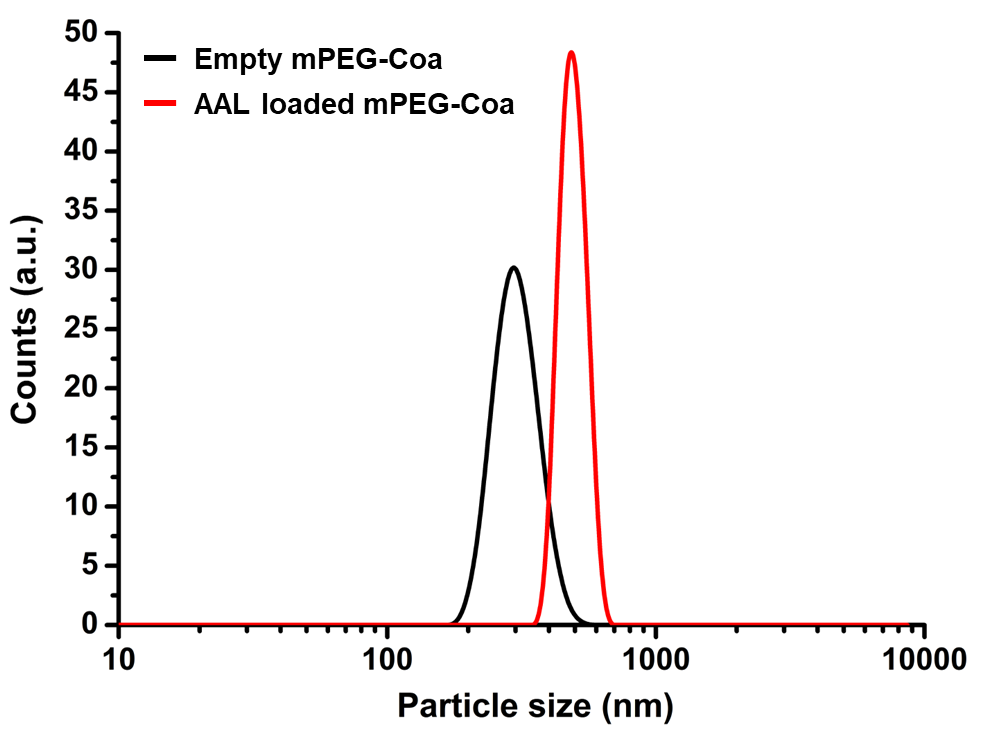
**

**Figure S1.** Size distribution of empty mPEG-Coa and AAL loaded mPEG-Coa.
